# Preparation and Characterization of Condensed Tannin Non-Isocyanate Polyurethane (NIPU) Rigid Foams by Ambient Temperature Blowing

**DOI:** 10.3390/polym12040750

**Published:** 2020-03-30

**Authors:** Xinyi Chen, Xuedong Xi, Antonio Pizzi, Emmanuel Fredon, Xiaojian Zhou, Jinxing Li, Christine Gerardin, Guanben Du

**Affiliations:** 1 LERMAB, University of Lorraine, 27 rue Philippe Seguin, 88000 Epinal, France; xinyi.chen@univ-lorraine.fr (X.C.); xuedong.xi@univ-lorraine.fr (X.X.); emmanuel.fredon@univ-lorraine.fr (E.F.); 2 Yunnan Key Laboratory of Wood Adhesives and Glue Products, Southwest Forestry University, Kunming 650224, China; xiaojianzhou1982@163.com (X.Z.); Jinxingli126@hotmail.com (J.L.); guanben@swfu.edu.cn (G.D.); 3 LERMAB, University of Lorraine, Boulevard des Aiguillettes, 54000 Nancy, France; christine.gerardin@univ-lorraine.fr

**Keywords:** mimosa tannin, rigid NIPU foam, self-blowing, MALDI-TOF, ^13^C NMR, FTIR

## Abstract

Ambient temperature self-blowing mimosa tannin-based non-isocyanate polyurethane (NIPU) rigid foam was produced, based on a formulation of tannin-based non-isocyanate polyurethane (NIPU) resin. A citric acid and glutaraldehyde mixture served as a blowing agent used to provide foaming energy and cross-link the tannin-derived products to synthesize the NIPU foams. Series of tannin-based NIPU foams containing a different amount of citric acid and glutaraldehyde were prepared. The reaction mechanism of tannin-based NIPU foams were investigated by Fourier Trasform InfraRed (FT-IR), Matrix Assisted Laser Desorption Ionization (MALDI-TOF) mass spectrometry, and ^13^C Nuclear Magnetic Resonance (^13^C NMR). The results indicated that urethane linkages were formed. The Tannin-based NIPU foams morphology including physical and mechanical properties were characterized by mechanical compression, by scanning electron microscopy (SEM), and thermogravimetric analysis (TGA). All the foams prepared showed a similar open-cell morphology. Nevertheless, the number of cell-wall pores decreased with increasing additions of glutaraldehyde, while bigger foam cells were obtained with increasing additions of citric acid. The compressive mechanical properties improved with the higher level of crosslinking at the higher amount of glutaraldehyde. Moreover, the TGA results showed that the tannin-based NIPU foams prepared had similar thermal stability, although one of them (T-Fs-7) presented the highest char production and residual matter, approaching 18.7% at 790 °C.

## 1. Introduction

Tannin, because of their distinct chemical properties, are classified into hydrolysable tannins and condensed or polyflavonoid tannins [1]. They are natural phenolic compounds, fairly ubiquitous in the vegetable world and commonly utilized as a starting materials in many fields, such as medicine [2,3], wastewater treatment [4,5,6], activated carbon [7,8], wood adhesives [9,10,11,12], fire resistance [13,14], coatings [15,16,17], etc. Among these applications, their use to prepare biobased foams for thermal and acoustic insulation and other applications has already attracted attention, especially in the case of condensed tannin [18,19,20]. Condensed tannins are mostly composed of polyhydroxy-flavan-3-ol oligomers, flavan-3,4-diols, and other flavonoid analogs linked by carbon-carbon bonds between flavonoid monomer units [1,21].

Several preparation approaches have been reported to produce tannin-based foams. The most used approach is by preparing tannin-furanic foams, obtained by the acid condensation of tannins and furfuryl alcohol [18,22,23,24,25,26], with foam expansion driven by a blowing agent activated by the temperature increase caused by the acid self-condensation of furfuryl alcohol. In such an approach, three-dimensional stabilization is achieved by adding a cross-linker, initially an aldehyde or other compounds [27,28,29]. Subsequently, to develop another kind of tannin-based foam or gel, other materials were utilized, such as amines [6,30], soy flour [27], lignin [20,28], polymeric diphenyl methane isocyanate (p-MDI) [30], etc. Using these raw materials mixed with different tannins did yield some tannin-based foam with better properties. In these methods, however, some of the chemicals added are either non-environmentally-friendly (volatile foaming agent) or have high prices. Therefore, mechanically blown tannin foam types were prepared as a novel preparation method [21,31,32]. This concept was inspired from the preparation of meringue from egg whites, tannin, or tannin-furan resin that were mixed with other ingredients, and then a large amount of air was introduced into the mixture by vigorous mechanical stirring, forming a liquid foam with a fast expansion speed [32]. Blowing agents were not needed in this approach, but the foams obtained had higher density and higher compressive strength than standard chemical foaming tannin-furan foams.

All the approaches described led to phenolic-type tannin or tannin-furanic foams. However, the greatest interest is still in polyurethane foams, in their preparation from mainly bio-based materials, and especially for non-isocyanate polyurethane foams (NIPU). Bio-based NIPU foams have already been prepared from a variety of other approaches and renewable raw materials [33,34,35,36,37,38,39,40,41,42,43,44,45,46,47,48]. Hence, based on the previous preparation methods and formulations, making use of some novel approaches and formulations to produce tannin-based foams did become one of the main targets of this research work. Moreover, while tannin-based NIPU resins have already been prepared for coatings or wood adhesives applications [49,50,51], tannin-based NIPU foams have never been reported. It is for these reasons that the present work deals with the preparation of self-blowing condensed tannin NIPU foams, the study of their morphology, their synthesis mechanism, their thermal stability, and their mechanical compression properties.

## 2. Materials and Methods 

### 2.1. Materials

Commercial mimosa tannin (Acacia mearnsii, De Wild) bark extract was obtained from Silva Chimica (St. Michele Mondovi, Italy). A 50% water solution of glutaraldehyde was obtained from Acros Organics (Geel, Belgium). Hexamethylenediamine (HDMA, 98%), Dimethyl carbonate (DMC, 99%, anhydrous), Hexamethylenetetramine (Hexamine, 99%, ACS reagent), and Citric acid (99.5%, ACS reagent) were supplied by Sigma-Aldrich (Saint Louis, France). All chemical reagents did not need purification before use.

### 2.2. Preparation of the Tannin-Based NIPU Resins

The method of tannin-based NIPU resin synthesis has been reported previously [52]. First, 40 g mimosa tannin was placed into a three-necked flask with condensing reflux condenser, a magnetic stirrer, and a thermometer. Then, 33.34 g of deionized water was added and stirred thoroughly. Second, 27 g of dimethyl carbonate (DMC) was added into the mixture, then mixed evenly, and heated to 65 °C, which keeps it at this temperature for 60 min. Third, 77.6 g of hexamethylene diamine (HDMA) (70% water solution) was added to the mixture, under continuous mechanical stirring, and heated to 90 °C, keeping it at this temperature for 120 min. Lastly, the resin obtained was collected and cooled down to room temperature, and ready for application.

### 2.3. Rigid Tannin-Based NIPU Foam Preparation

Foam formulations were prepared using the amounts of reagents listed in Table 1. The foams were obtained by mixing two compounds. The first one is a homogeneous acid mixture, the composition of which consists of citric acid (50% in water solution) and glutaraldehyde (50% in water solution). The second compound is a homogeneous tannin NIPU resin, composed of the mimosa tannin-based resin and hexamine. Briefly, different dosages of tannin-based resins (as referred) and hexamine were added into plastic cups, and stirred rapidly, which resulted in homogeneous tannin resins ready for application. The mixture of citric acid and glutaraldehyde was weighed and put into a foaming module, and, then, the tannin-based resin and hexamine mix was added immediately, while stirring manually for an optimal predetermined period of 10–15 s. Subsequently, the foams were left to grow at ambient temperature (25 °C). When the self-blowing step was finished, a homogeneous dark and red liquid self-supporting foam was obtained. Thus, it was by necessity cured at 70–80 °C overnight to obtain the final rigid foam. Lastly, the hardened foam samples were conditioned for a minimum of two days at 25 °C and 12% relative humidity before being characterized.

### 2.4. Apparent Density

According to the standard method of ASTM D1622-08, all testing foam samples were prepared to a size of 30 mm × 30 mm × 30 mm. The ratio of weight to cubic volume of the specimen volume was defined as density. Five sample repeats were tested for each foam.

### 2.5. Scanning Electron Microscopy (SEM) Analysis

Scanning electron microscopy (SEM, Hitachi TM-3000)(Milexia, Paris, France) was used to analyze the microstructure and morphology of the foams obtained. All samples were made into 0.5 cm^2^ (cross section). Then, a thin layer of gold-palladium was sputtered on the surface of the foams so that a better definition is obtained.

### 2.6. Fourier Transform Infrared (FT-IR) Spectroscopy

PerkinElmer Frontier ATR-FT-MIR (PerkinElmer, Villebon-sur-Yvette, France) was used to investigate the functional groups of all foams. The sample powder was placed in a 1.8-mm diamond eye of the Attenuated Total Refection Fourier Transform InfraRed (ATR-FT-MIR) equipment. In addition, 32 scans at a resolution of 4 cm^−1^ were done for each sample between 600 and 4000 cm^−1^.

### 2.7. MALDI-TOF

A total of 5 mg of sample powder were dissolved in 1 mL of a 50:50 *v*/*v* acetone/water solution. Then, 10 μL of a 2,5-dihydroxy benzoic acid (DHB) matrix was added to 10 mg of the sample solution. Furthermore, 2 μL of an NaCl solution 0.1 M in 2:1 *v*/*v* methanol/water were applied and pre-dried on the sample support plaque, which is followed by the addition of 1 μL of the sample solution. The plaque was then dried again. The standardization of the MALDI spectrometer was done with red phosphorous. The spectrometer used was an Axima-Performance from Shimadzu Biotech (Kratos Analytical Shimadzu Europe Ltd., Manchester, UK). The tuning mode was linear polarity-positive. A total of 1000 transients for each sample were done with two shots accumulated per profile. The spectrum precision is of ±1 Da.

### 2.8. Solid State CP MAS ^13^C NMR

Solid state Cross Polarisation-Magic Angle Spinning CP MAS ^13^C NMR was used to analyze the cured foam powder. The spectromer used was an AVANCE II 400 MHz spectrometer (Brüker, Billerica, MA, USA). Furthermore, 100.6 MHz was the frequency used at a 12-kHz sample spin, and the recycling delay was 1 s, depending on the 1H spin lattice relaxation times (t1) estimated with the inversion-recovery pulse sequence, and a contact time of 1 ms. The decoupling field was 78 kHz with 15,000 being the number of transients. Tetramethylsilane (TMS) was used as the shift control. The spectra precision was of ±1 ppm. Spinning side bands suppression was used.

### 2.9. Compression Resistance

The samples were cut into a uniform size of 25 mm × 25 mm × 25 mm. A universal testing machine (Instron 3300, Elancourt, France) was used to test the compression strength of the foams. The direction of load was parallel to that of the foam rise under ambient conditions. The crosshead rate was fixed at 2.0 mm·min^−1^. At least three sample repeats were tested for each foam.

### 2.10. Thermogravimetric Analysis (TGA)

A TGA5500 analyzer (Mettler Toledo, Guyancourt, France) was used to measure the foam’s thermal stability. Anywhere from 5 to 8 mg sample powder was placed on the platinum pan, and then heated with the sample at a temperature rate of 10 °C·min^−1^, under a nitrogen atmosphere (50 mL/min). The temperature range used was 25 °C to 790 °C.

## 3. Results and Discussion

### 3.1. Preparation of Tannin-Based NIPU Foams

**Scheme 1:**
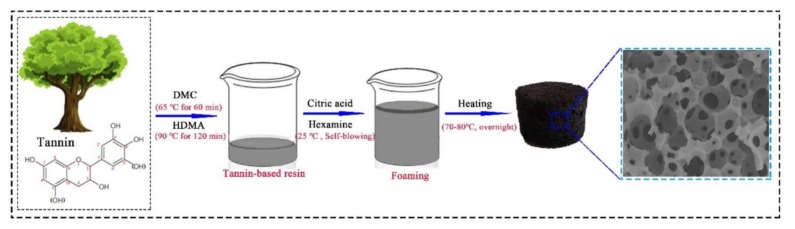
The whole preparation process of a tannin-based NIPU foam.

**Scheme 2:**
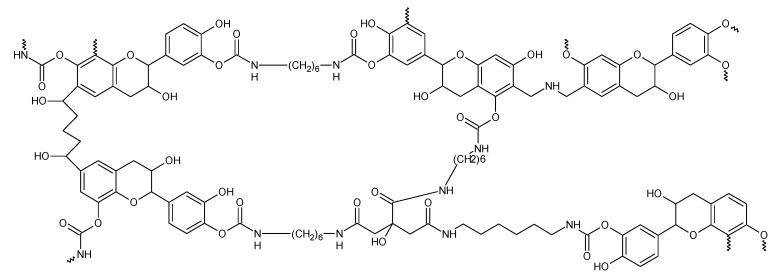
A schematic example of some of the mixed linkages present in a possible network structure of tannin-based NIPU foam.

The tannin-based NIPU foams were prepared by a self-blowing approach. Because foaming and cross-linking occur almost simultaneously, they could not be strictly separated into two processes occurring independently. However, to investigate the process, the process can be assumed as two main separate steps: one is the foaming and the other one is the cross-linking. The whole preparation process of a tannin-based NIPU foam is shown in Scheme 1. The self-blowing energy for foaming (cf. Scheme 1) comes from the reaction of citric acid and the amino groups of both the hexamine and possibly with some still free amino groups of the tannin NIPU resin, resulting in the volume expansion of the liquid foams [53]. Glutaraldehyde functions as cross-linker, ensuring that the liquid foams system does not collapse and maintains itself self-supporting. Again, glutaraldehyde cross-links by reacting with the amino groups and with the reactive aromatic ring sites of the tannins [53], which contributes to form the three-dimensional structure of the tannin-based NIPU foam. Furthermore, hexamine participates to cross-linking by forming bridges between tannin molecules during the periods of foaming and heating. It must be noted that the liquid foams could not be maintained without collapsing when not adding the cross-linkers, i.e., hexamine or glutaraldehyde. This conclusion was confirmed in preparation experiments (cf. Table 2, where for T-Fs-5 and T-Fs-9, no foam samples were obtained). Therefore, the cross-linking process is a critical step for maintaining the three-dimensional structure of the liquid tannin-based NIPU foams obtained. Ultimately, this double effect causes the volume expansion, the sharp increase of the viscosity of the mixture, and then its gelling, which results in a three-dimensional network. A schematic example of some of the mixed linkages present in a possible network structure of tannin foams is shown in Scheme 2.

### 3.2. Apparent Density

**Figure 1:**
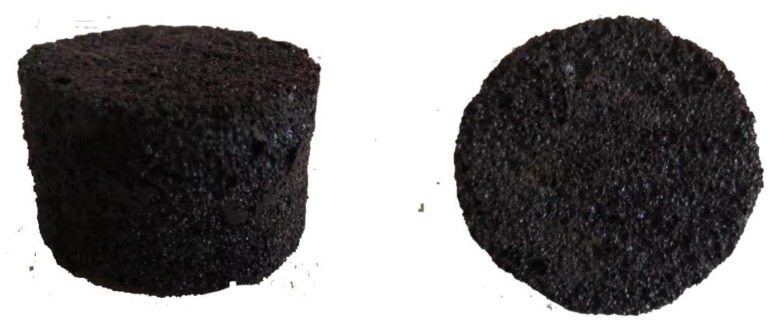
Digital photo of tannin-based NIPU foam.

The self-blowing process can provide a flexible liquid foam with a three-dimensional network, which needs to be hardened by heating so that the necessary strength for measuring can be obtained. As shown in Figure 1, tannin-based NIPU foams with stable properties were prepared. The black foam with slightly red coloring is due to the presence of tannin [54]. The apparent densities of the foams are shown in Table 2. The foams apparent densities increase with the increasing proportion of glutaraldehyde. The maximum foams apparent density approximates 0.26 g·cm^−3^ for the addition of 4 g of glutaraldehyde. The reason for such an increase is the high reactivity of glutaraldehyde, which bridges two tannin oligomers with each other or with amino groups. It causes a rapid gel, which results in a higher cross-linking level much earlier in the foaming process. Furthermore, the relationship of citric acid with the foams’ apparent density was also investigated. Table 2 shows that the addition of citric acid can slightly affect the foams’ apparent density. Comparing T-Fs-2 with T-Fs-11 shows that the foam apparent density decreases slightly with the increasing amount of citric acid, from 0.15 g·cm^−3^ to 0.12 g·cm^−3^. This is attributed to the foaming energy originating from the reaction of citric acid with the –NH_2_ groups [53]. Thus, a larger expansion volume can be obtained by increasing the amount of citric acid to obtain a smaller apparent density.

### 3.3. Scanning Electron Microscopy (SEM) Analysis

**Figure 2:**
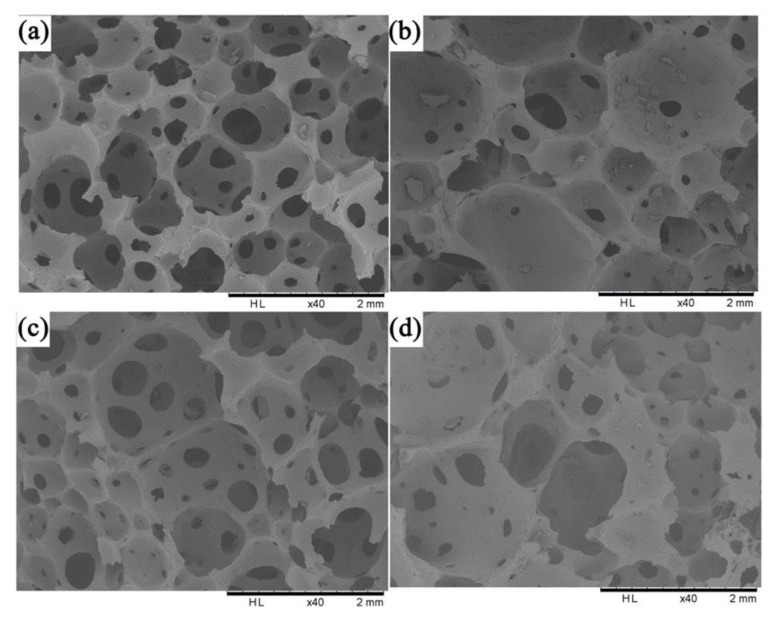
SEM images of tannin-based NIPU foams. (**a**) T-Fs-2, (**b**) T-Fs-7, (**c**) T-Fs-11, and (**d**) T-Fs-13.

Scanning electron microscopy (SEM) observation was utilized to investigate the morphology and microscopic structure of different foam formulations. The SEM images of foam samples are shown in Figure 2. It shows that all these foams present an open-cell structure. A considerable number of open pores are observed in the SEM images, which are attributed to water evaporation in the precursor resins during foaming and drying. Furthermore, some ruptures or debris and incomplete cellular structures can be seen in all foam samples, with these being due to the cutting process for preparing the samples [54].

Comparing Figure 2a (2 g glutaraldehyde) and Figure 2b (4 g glutaraldehyde), although they present a similar morphology and microscopic structure, less perforations are clearly apparent in T-Fs-7. The most likely explanation of this is that the high reactivity of glutaraldehyde with the tannin in the NIPU resin promotes the formation of higher molecular weight macromolecules, which causes a more rapid and more marked increase in the viscosity of the foaming system. Consequently, this weakens the pore-creating effect of water evaporation during foaming and drying. This effect can still be clearly observed even at a high amount of citric acid. Figure 2d also shows fewer cell perforations than in Figure 2a. Equally, as the volume expansion of the liquid foam is hindered by the high viscosity, this eventually results in a high-density foam. This conclusion is confirmed by the results in Table 2.

Thus, it appears that the dual function of citric acid is to effectively influence both foam morphology and its microscopic structure. This can be seen when a larger proportion of citric acid was used, as shown in Figure 2c (8 g of citric acid) and Figure 2d (9 g of citric acid). The reason for this might be that more citric acid can react with amino groups to provide more energy for foaming, which leads to a better volume expansion of the liquid foam [53]. Simultaneously, this resulted in a smaller foam density, as shown in Table 2. Thus, citric acid not only appears to provide more energy for foaming but also appears to contribute to strengthen and stabilize the three-dimensional foam structure.

### 3.4. The Reaction Mechanism of Tannin-Based NIPU Foams

#### 3.4.1. Fourier Transform Infrared (FT-IR) Spectroscopy

**Figure 3:**
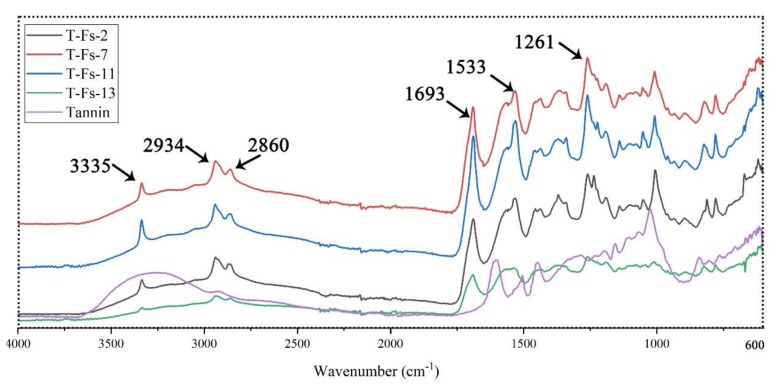
FT-IR spectra of tannin-based NIPU foams.

Fourier transform infrared (FT-IR) spectra of neat mimosa tannin and, of the NIPU foams, are shown in Figure 3, to investigate the functional groups changes occurring in these foam preparations. The results indicate that some significant variation can be found between mimosa tannin and the NIPU foams obtained from it. The FT-IR spectra curves of all foam samples present similarities with each other. The infrared absorption spectrum of the mimosa tannin and of the NIPU foams show an intense band between 3500 and 3100 cm^−1^ attributed to the –OH stretching vibration [55,56]. Moreover, a band at 3337 cm^−1^ in all NIPU foams spectra is assigned to –N–H stretching of the NIPU urethane linkages derived from the reaction of the tannin with DMC and HDMA [50]. Citric acid reacts with –NH_2_ groups to produce amides as well. There are two bands at 2934 cm^−1^ and 2860 cm^−1^, relative to the C–H stretching vibration of –CH_2_ and –CH_3_ [56,57]. These two bands are not detected in mimosa tannin, which indicates some functional groups have been changed during the preparation of the tannin-based resin and the foaming. Furthermore, present in the foams but not in the tannin, are the band at 1693 cm^−1^ assigned to a C=O and that confirms its assignment and the assignment of the 3337 cm^−1^ bands to belong to a urethane linkage [52]. Moreover, the final confirmation of the urethane linkage is the presence of its other characteristic band at 1533 cm^−1^ (cf. Scheme 1 adducts 1). In addition, the band at 1261 cm^−1^ is representative of amines C–N elongation [50].

#### 3.4.2. MALDI-TOF Analysis

**Figure 4:**
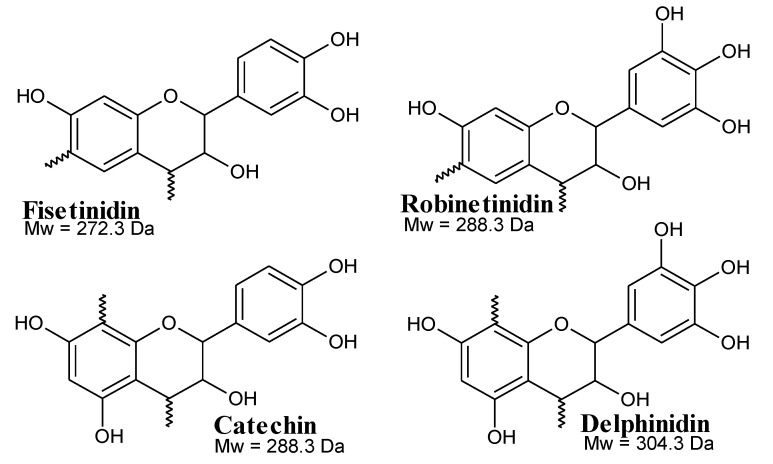
The four main structures of flavonoid units of the condensed tannin used.

**Figure 5:**
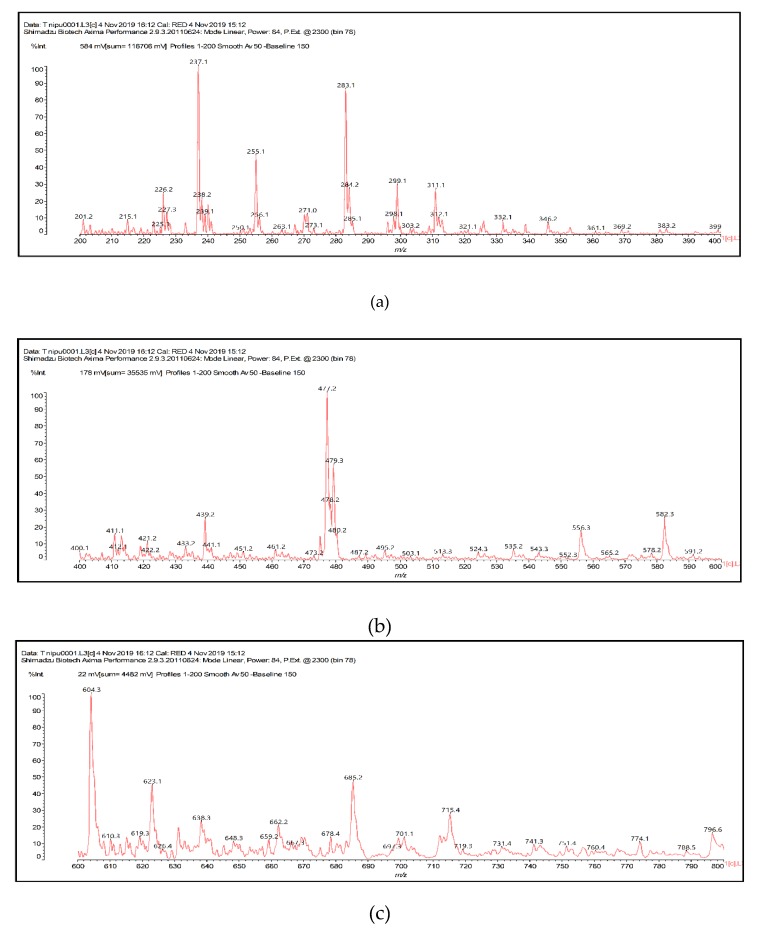

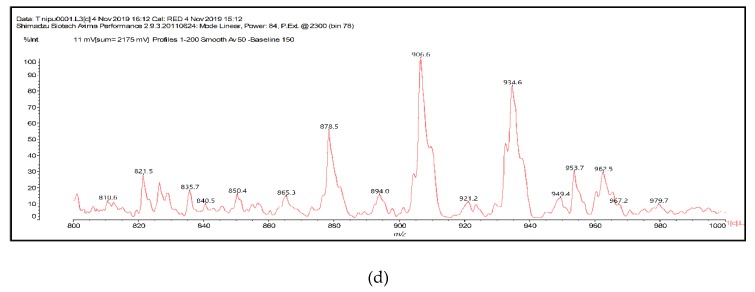
MALDI-TOF spectra of tannin-based NIPU foam (T-Fs-3), (**a**) 200–400 Da range, (**b**) 400–600 Da range, (**c**) 600–800 Da range, and (**d**) 800–1000 Da rang.

To determine the distribution of condensed tannin oligomers and its derived condensation products, MALDI-TOF mass spectrometry has been used. The basic flavonoid units composing the tannin used are shown in Figure 4. The MALDI-TOF spectra of tannin-based NIPU foam T-Fs-7 is shown in Figure 5. Furthermore, some foreseeable species in the preparation of the co-reaction of tannin-based biomass foam are shown in Table 3. In general, as shown in Figure 4, four types of flavonoid units are involved in the formation of condensed tannin-derived oligomers, i.e., Fisetinidin, Robinetinidin, Catechin, and Delphinidin, respectively [24,50,58]. The tannin-derived products will originate from the combination of these units with each other and with other reagents. In view of Figure 5a, unreacted flavonoid oligomers (+23 Da from Na^+^) of condensed tannin monomers can be seen, i.e., 298–299 Da, 311–312 Da, and 326.2 Da.

**Scheme 3:**
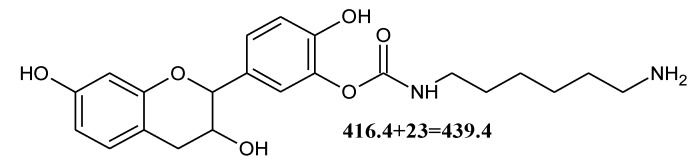
One of the possible structures of the urethane between a flavonoid unit, DMC, and HDMA.

Furthermore, some peaks in Figure 5 show that the tannin-derived NIPUs are obtained during the tannin-based NIPU resin preparation [49,51]. Thus, the peaks at 433.2 Da, 439.2 Da, 451.8 Da, and 556.3 Da shown in Figure 5b, are assigned tannin-based NIPU oligomers (cf. Scheme 3) and all have already been reported [49,50,51]. This kind of tannin-derived oligomers (Tannin-based non-isocyanate polyurethanes, recorded as tannin-based NIPU) can be recognized by the urethane bonds (–NH–CO–O–), which support and confirm the FT-IR and solid state ^13^C NMR findings. Higher NIPU tannin oligomers can also be formed.

**Scheme 4:**
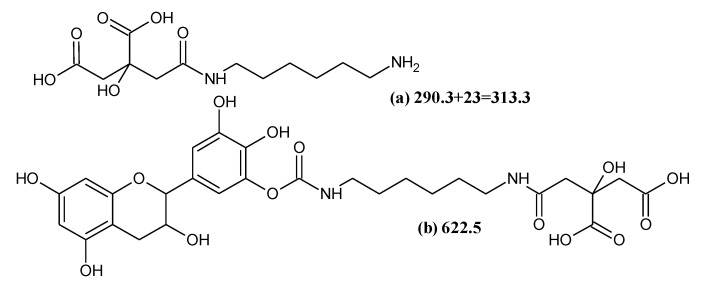
Two kinds of citric acid derived products: (**a**) –NH–CO– bond containing molecules and (**b**) both –NH–CO– and –NH–CO–O– bonds containing molecules.

On account of the high reactivity between citric acid and –NH_2_ groups (HDMA or tannins-based NIPU), the possible reaction products can be detected. This series of reactions is also the source of energy for foam volume expansion [53]. These peaks are such as 313.1 Da in Figure 5a, 411.1 Da in Figure 5b, 604.3 Da, 615.4 Da, 619.5 Da, and 741.5 Da in Figure 5c, and 828.6 Da, 865.2 Da, 878.6 Da, 906.6 Da, 921.5 Da, 954.5 Da, and 963.4 Da in Figure 5d. Among all these, they are reaction combination products based on citric acid (cf. Scheme 4). Ultimately, an amide bond (–NH–CO–) can be formed with citric acid in these derived products. Moreover, there are two types of these products derived from the reactions of citric acid, according to whether they do or do not include tannin-based NIPU, i.e., urethane bonds (–NH–CO–O–). Examples of these two types are shown in Scheme 4.

**Scheme 5:**
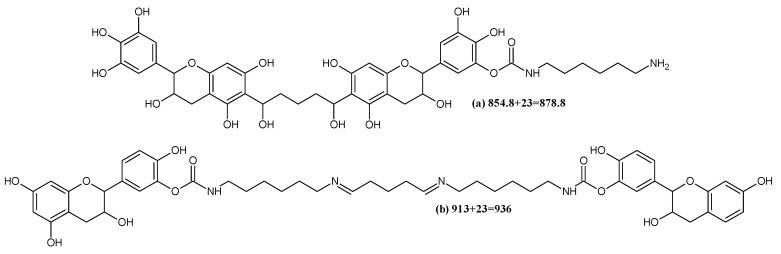
Examples of two kinds of glutaraldehyde derived products: (**a**) glutaraldehyde bridging directly two aromatic tannin rings, and (**b**) glutaraldehyde bridging oligomers presenting –NH_2_ groups.

Glutaraldehyde forms bridges and crosslinks connecting two or more oligomers to yield larger molecular weight products [53]. Glutaraldehyde can react with ease with tannin-derived NIPUs and other –NH_2_ groups. The foreseeable cross-linked oligomers are shown in Scheme 5. Thus, glutaraldehyde either links directly with the aromatic rings of tannin units or with the compounds presenting –NH_2_ groups. Examples of the cases as shown in Scheme 5 are represented by the peaks at 649.3 Da, 662.2 Da, 683.4 Da, 699.4 Da, and 715.4 Da in Figure 5c, 821.5 Da, 830.1 Da, 878.6 Da, 894.4 (921.5) Da, 934.9 Da, and 951 Da in Figure 5d. Even a new cross-link –C=N– bond can occur in these products.

**Scheme 6:**
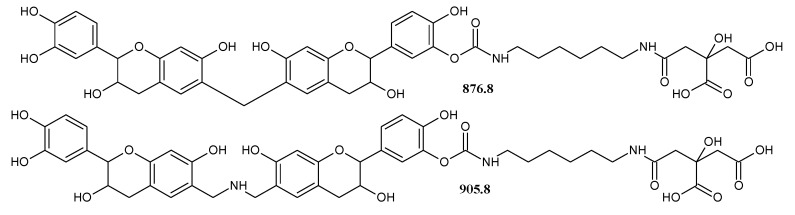
Example of foreseeable structures of tannin-hexamine reaction products.

Hexamethylenetetramine (hexamine) has been commonly used as a hardener of tannin-based wood adhesives and foams, as already reported [59,60]. Without doubt, it can also play a positive role in crosslinking and curing the liquid foams. Moreover, while tannins and hexamine have a complex polymerization reaction mechanism, two types of linkages can still be distinguished [61,62,63], namely two kinds of methylene-based reactive fragments derived from hexamine can serve as cross-linkers: –CH_2_–NH–CH_2_– and N–(CH_2_)_3_–. Typical tannin-hexamine structures are shown in Scheme 6. From Figure 5, the MALDI-TOF spectra indicate some evidence of this, including the peaks at 578.3 Da, 582.4 Da, 591.2 Da in Figure 5b, 604.3 Da, 611.3 Da, 615.4 Da, 619.5 Da, 623.2 Da, 631.3 Da, 638.4 Da, 768.7 Da, and 796.5 Da in Figure 5c, and 878.6 Da, 906.6 Da, 954.5 Da, and 963.4 Da in Figure 5d. They are assigned to some tannin-hexamine reaction products.

#### 3.4.3. Solid State CP MAS ^13^C NMR Analysis

**Figure 6:**
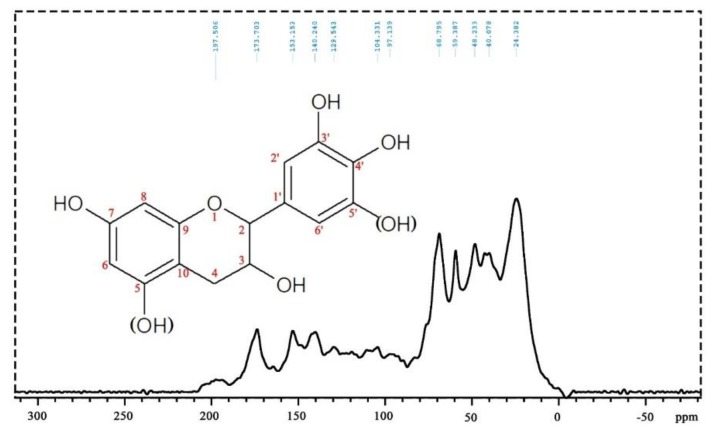
Solid state ^13^C NMR spectrum of tannin-based NIPU foam (T-Fs-7).

Solid state Cross Polarization Magic Angle Spinning (CP MAS) ^13^C NMR is a useful technique to investigate the composition of the foams prepared [24,64,65]. The ^13^C NMR spectrum of a tannin-based NIPU foam (T-Fs-7) is shown in Figure 6. Several peaks can be observed. First of all, the peaks belonging to the tannins are relatively small. One can, thus, distinguish the shoulder at 157 ppm of the tannin C5 and C7, the wide peak at 153–155 ppm both belonging to the C9 of flavonoid units, and possibly the C=O of a urethane linkage does contribute [24]. The small peak/shoulder at 150 ppm more clearly belongs to a urethane linkage. This belonging to the aromatic ring carbon linked to a urethane linkage is of the type shown below. 
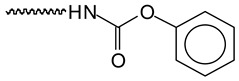
 This supports the indication that urethane linkages on the aromatic tannin rings have formed and subsist in the final foam network. The peak at 48 ppm is assigned to the –CH_2_– of glutaraldehyde next to an unreacted aldehyde group. An overlapping peak does appear at 40 ppm and presents two little peaks that can be seen in this position. The peak at 40 ppm and the shoulder at 38 ppm belong to the –CH_2_– next to the aldehyde group that has reacted with the tannin aromatic ring site. The other one possible explanation is that it belongs to –CH_2_– on the unreacted heterocycle C4 site of the flavonoid [64]. This is, however, unlikely, considering the intensity of the signal in relation to the low intensity of all the other signals of the flavonoid carbons. The more likely explanation is that it is likely to belong to the inner –CH_2_– groups of glutaraldehyde or citric acid.

The huge and broad peak at 24 ppm is assigned to the sum of –CH_2_– groups of hexamethylenediamine and the remaining ones of glutaraldehyde. The peak at 69 ppm is assigned to the C–OH generated by the reaction of aldehyde groups of glutaraldehyde with an aromatic ring site of the tannin and masks the C3 signal of flavonoid units. The peak at 173 ppm and the shoulder at 175 ppm belong to the C=O groups of citric acid under two different environments. One is unreacted, and the other reacted to form a –CO–NH– bond derived from the reaction of the acid with compounds carrying –NH_2_ groups. The wide series of peaks at 197–203 ppm are assigned to the –CHO group of glutaraldehyde. Regarding the flavonoid units, the peak at 143–145 ppm belongs to the C3′, C4′, and C5′ of the flavonoid B-ring, the peak at 130–131 ppm to the C1′, the small peak at 120 ppm to the C6′, and the peaks at 110 ppm and 115 ppm to the two types of interflavonoid oligomer bond C4, C8 and C4, C6. The small peak at 82 ppm belongs to the flavonoid C2. The marked peaks at 59 ppm belongs to the carbohydrates that are present in the industrial tannin extract used.

Thus, it is confirmed that the condensed tannin-based NIPU has been synthesized by combining the tannin with DMC and HDMA in the tannin-based resin preparation stage. A clear and marked shoulder peak at 173.7 ppm is that of the –CO–NH– bond, which exist in plenty of compounds in the foam rooted from the reaction citric acid with –NH_2_ containing molecules. The more likely explanation is that it is likely to belong to the inner –CH_2_– groups of glutaraldehyde or citric acid.

### 3.5. Compression Mechanical Properties

**Figure 7:**
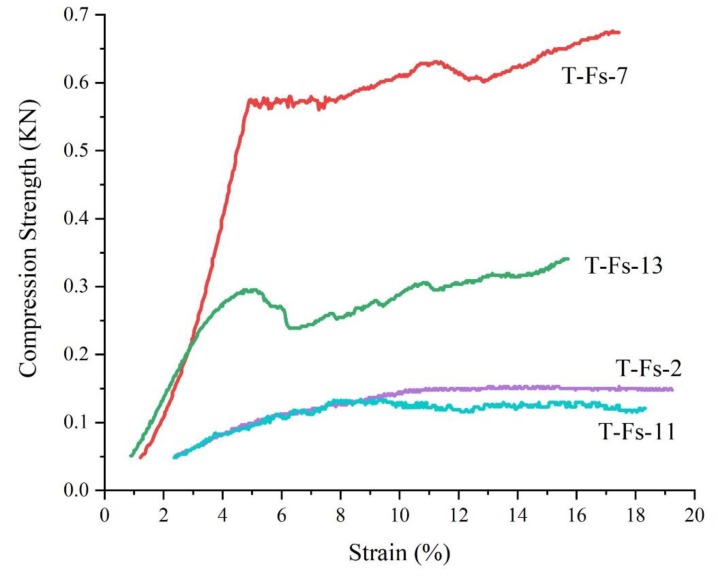
Compressive stress-strain curves of T-Fs-2, T-Fs-7, T-Fs-11, and T-Fs-13.

The compression stress-strain curves of T-Fs-2, T-Fs-7, T-Fs-11, and T-Fs-13 samples are shown in Figure 7. Intuitively, T-Fs-7 exhibits the highest compression strength, for 0.57 kN, and then T-Fs-13 for 0.32 kN, T-Fs-2 for 0.15 kN, and T-Fs-11 for 0.12 kN, respectively. The reason for this is attributed to the apparent density of the foams with this being directly proportional to compression strength [53]. This conclusion is in line with previous works indicating that higher density leads to higher strength [18,53,66]. In addition, lower density foams have a thinner cell wall [18,54]. Theoretically, thinner cell walls can only provide a rather limited contribution to compression resistance. Thus, eliminating the influence of the foam’s density, the specific compression strength was evaluated according to the literature [66]. The results of specific compression strength for all foam samples are shown in Table 2. For T-Fs-2, T-Fs-7, T-Fs-11, and T-Fs-13 samples, the corresponding specific compressive strengths are 1.62 kPa/kg·m^−3^, 3.47 kPa/kg·m^−3^, 1.63 kPa/kg·m^−3^, and 2.31 kPa/kg·m^−3^, respectively. Their trend is clearly in line with the apparent foam’s densities. The conclusion of this is that the strength trend is not only exclusively attributed to the foam density alone, but also related to the contribution of the cell wall. Furthermore, another possible explanation is the dependence on the number of cell wall perforations, which can break the structural integrity of the cell. Therefore, this contributes to the decrease of compression strength. This conclusion is also supported by the combination of Figure 2 and Table 2. In brief, the improvement of mechanical properties of tannin-based biomass foams is a result of a multi-factor synergy.

### 3.6. Thermogravimetric Analysis (TGA)

**Figure 8:**
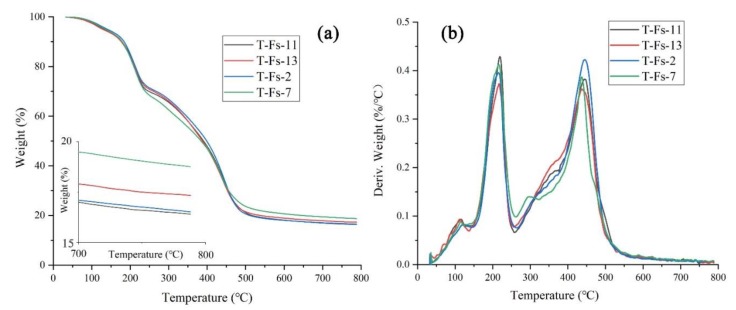
TGA (**a**) and Derivative Thermogravimetry (DTG) (**b**) curves of T-Fs-2, T-Fs-7, T-Fs-11, and T-Fs-13 (under N_2_ atmosphere).

To evaluate the thermal stability of tannin-based biomass foams, the thermogravimetric analysis (TGA) curves of T-Fs-2, T-Fs-7, T-Fs-11, and T-Fs-13 are shown in Figure 8. The corresponding specific degradation temperatures and char yields at 790 °C are listed in Table 4. The *T_max_* value reported in Table 4 is the maximum temperature shown by DTG curve peaks at different pyrolysis stages. A three-stage similar pyrolysis behavior of tannin-based NIPU foams are observed in Figure 8. The initial weight loss occurs within the temperature range of 30 to 150 °C with this being related to the decomposition of the excess acid and hexamine and the release of the volatilized absorbed water [54]. In this step, 3.3% of weight loss occurred for T-Fs-2 and T-Fs-7 while 3.6% of weight loss occurred for T-Fs-11 and T-Fs-13. The second weight loss range is between 150 °C and 250 °C, showing 18.3% of weight loss for T-Fs-2, 19.2% of weight loss for T-Fs-7, 19.3% of weight loss for T-Fs-11, and 18.1% of weight loss for T-Fs-13. The weight loss in this range is related to decomposition reactions by bond cleavage of urethane and tannin intermolecular bonds (onset temperature of mimosa tannin was 146 °C) [55,67]. The third weight loss occurs in the 350 °C and 550 °C range. This is the stage where the largest weight mass loss occurs, which is larger than 40%. Thus, in this temperature range, 44.1% of weight loss for T-Fs-2, 41.2% of weight loss for T-Fs-7, 43.7% of weight loss for T-Fs-11, and 42.9% of weight loss for T-Fs-13 take place. This step may be caused by breaking C–C bonds and the decomposition of pyrolysis residual products from the first two stages [55,67]. These results show that the tannin-based NIPU foams present similar pyrolysis temperature weight losses (within 8 °C). Nevertheless, a slight difference in the residual mass at 790 °C of the tannin-based NIPU foams occurs, according to a predictable trend. The residual masses of 16.5% for T-Fs-2 and T-Fs-11, 17.3% for T-Fs-13, and 18.7% for T-Fs-13 show an increasing relation with the higher proportion of glutaraldehyde addition. This is due to a better cross-linked three-dimensional foam system due to the increasing addition of glutaraldehyde.

## 4. Conclusions

The work presented in this case reports a novel mimosa tannin-based NIPU rigid foam using ambient temperature self-expansion to cause the foaming. An acid mixture blowing agent, including citric acid and glutaraldehyde, was used to provide the foaming energy and cross-link the tannin-derived products to produce self-supporting tannin-based NIPU foams without needing any volatile blowing agents. Four types of tannin-based foams were prepared by using different proportions of citric acid and glutaraldehyde. FT-IR, MALDI-TOF, and ^13^C NMR contributed to the analysis of the reaction mechanism and products formed indicating, among others, that urethane linkages were formed. Furthermore, SEM images exhibit similar open-cell morphology. The number of cell-wall pores decreased with increasing additions of glutaraldehyde, while bigger foam cells were obtained with the increasing addition of citric acid. The compressive mechanical properties were enhanced by the improved level of cross-linking between tannin NIPU molecules at higher amounts of glutaraldehyde. Thermogravimetric analysis (TGA) results showed that T-Fs-7 presented the highest char production, approaching a residual 18.7% at 790 °C under nitrogen atmosphere.

## Figures and Tables

**Table 1:** The formulation compounds of tannin-based NIPU foams.

Foams	Resins (g)	Hexamine (g)	Citric Acid (g)	Glutaraldehyde (g)
T-Fs-2	10	2	6	2
T-Fs-5	10	0	6	2
T-Fs-7	10	2	6	4
T-Fs-9	10	2	4	0
T-Fs-11	10	2	8	2
T-Fs-13	10	2	9	3

**Table 2:** Apparent density, compressive strength, and specific compressive strength of tannin-based NIPU foams.

Foams	Apparent Density (g/cm^3^)	Compressive Strength (KN)	Specific Compressive Strength (kPa/kg·m^−3^)
T-Fs-2	0.15 ± 0.02	0.15 ± 0.02	1.62 ± 0.33
T-Fs-5	-	-	-
T-Fs-7	0.26 ± 0.04	0.57 ± 0.03	3.47 ± 0.38
T-Fs-9	-	-	-
T-Fs-11	0.12 ± 0.03	0.13 ± 0.01	1.63 ± 0.24
T-Fs-13	0.22 ± 0.02	0.32 ± 0.03	2.31 ± 0.18

**Table 3:** MALDI-TOF interpretation of species obtained in the co-reaction of a tannin-based NIPU foam (Fisetinidin—Fi; Robinetinidin—Ro; Delphinidin—De). Note: the MW of Catechin and Robinetinidin are the same. Whenever Ro is indicated, it could well be Catechin. Since the relative abundance of Robinetinidin is much higher in mimosa tannin, Ro is always indicated in this table.

Peak (Da)	Experimental Peak (Da)	Species
299.1	274.2	Fi
311.1	290.2 + 23 = 313.2	Ro with Na^+^
313.1	290.3 + 23 = 313.3	Citric acid-HDMA
326.2	306.2 + 23 = 329.2	De with Na^+^
433.2	432.4	Ro-DMC-HDMA
411.1	388.5 + 23 = 411.5	Citric acid-(HDMA)_2_ with Na^+^
439.3	416.4 + 23 = 439.4	Fi-DMC-HDMA with Na^+^
451.2	448.4	De-DMC-HDMA
556.3	558.6	Fi-(DMC)_2_-2HDMA
578.3	576.5	Fi-CH_2_-Ro
582.4	560.5 + 23 = 583.5	Fi-CH_2_-Fi with Na^+^
591.3	592.5	Ro-CH_2_-Ro
604.3	606.5 and/or 605.5	Ro-DMC-HDMA-Citric and/or Ro-CH_2_NHCH_2_-Fi
615.4	590.6 + 23 = 613.6 and/or 590.5 + 23 = 612.5 and/or 592.5 + 23 = 615.5	De-(DMC)_2_-(HDMA)_2_ and/or Fi-DMC-HDMA-Citric and/or Ro-CH_2_-Ro with Na^+^
611.3	589.5 + 23 = 612.5	Fi-CH_2_NHCH_2_-Fi with Na^+^
619.5	621.5 and/or 622.5	De-DMC-HDMA-Citric and/or Ro-CH_2_NHCH_2_-Ro
623.2	624.5	De-CH_2_-De
631.3	608.5 + 23 = 631.5	Ro-CH_2_-De with Na^+^
638.4	637.5	Ro-CH_2_NHCH_2_-De
649.3	648.6	Fi-CH(–OH)–(CH_2_)_3_–CH(–OH)-Fi
662.2	664.6	Ro-CH(–OH)–(CH_2_)_3_–CH(–OH)-Fi
683.4	680.6	Ro-CH(–OH)–(CH_2_)_3_–CH(–OH)-Ro
699.4	696.6	De-CH(–OH)–(CH_2_)_3_–CH(–OH)-Ro
715.4	712.6	De-CH(–OH)–(CH_2_)_3_–CH(–OH)-De
741.5	720.7 + 23 = 743.7	De-DMC-(HDMA)_2_-Citric with Na^+^
768.7	747.7 + 23 = 770.7	Fi-CH_2_NHCH_2_-Ro-DMC-HDMA with Na^+^
796.5	795.7	De-CH_2_NHCH_2_-Ro-DMC-HDMA
828.6	830.9	Fi-(DMC)_2_-(HDMA)_3_-Citric
821.6	822.8	Ro-CH(–OH)(CH_2_)_3_CH(–OH)-Ro-DMC-HDMA
830.1	806.8 + 23 = 829.8	Fi-CH(–OH)(CH_2_)_3_CH(–OH)-Ro-DMC-HDMA with Na^+^
865.2	862.9	De-(DMC)_2_-(HDMA)_3_-Citric
878.6	854.8 + 23 = 877.8 and/or 876.8	De-CH(–OH)(CH_2_)_3_CH(–OH)-De-DMC-HDMA with Na^+^, and/or Fi-CH_2_-Fi-DMC-HDMA-Citric
894.4 (921.5)	897 and/or 897 + 23 = 920	Fi-DMC-HDMA-CH(–OH)(CH_2_)_3_CH(–OH)-Fi without and/or with Na^+^ (920 Da)
906.6	905.8	Fi-CH_2_NHCH_2_-Fi-DMC-HDMA-Citric and/or Ro-CH_2_NHCH_2_-Ro-CH_2_-Fi
921.5	921.8	Ro-CH_2_NHCH_2_-Fi-DMC-HDMA-Citric and/or Ro-CH_2_NHCH_2_-Ro-CH_2_-Ro and/or De-CH_2_NHCH_2_-Ro-CH_2_-Fi
934.9	913 + 23 = 936	Ro-DMC-HDMA-CH(–OH)(CH_2_)_3_CH(–OH)-Fi-DMC-HDMA with Na^+^
951	829 + 23 = 952	Ro-DMC-HDMA-CH(–OH)(CH_2_)_3_CH(–OH)-Ro-DMC-HDMA with Na^+^
954.5	953.8	De-CH_2_NHCH_2_-Ro-DMC-HDMA-Citric
963.4	937.8 + 23 = 960.8	Ro-CH_2_NHCH_2_-Ro-DMC-HDMA-Citric with Na^+^

**Table 4:** TGA data of T-Fs-2, T-Fs-7, T-Fs-11, and T-Fs-13 (under nitrogen atmosphere).

Samples	*T_max_* (°C)			Residual Mass at 790 °C (%)
	Step Ⅰ	Step Ⅱ	Step Ⅲ	
T-Fs-2	119.2	214.8	444.8	16.5
T-Fs-7	115.1	215.4	436.2	18.7
T-Fs-11	114.8	218.6	445.4	16.4
T-Fs-13	112.2	216.1	437.1	17.3
